# 

**Published:** 1833-04-01

**Authors:** 


### CONTENTS

OF THE

### MEDICO-CHIRURGICAL REVIEW,

No. XXXVI. APRIL 1, 1833,

### REVIEWS.

I.

Leçons Orales, &c.—Clinical Lectures in Surgery, delivered at the Hôtel Dieu, Paris. By Baron DUPUYTREN. Vol. the First, Paris, 1832. .. .. .. .. 289

ART. I. Permanent Retraction of the Fingers.. .. .. .. .. .. 290

II. On Cataract .. .. .. .. .. .. .. .. .. .. .. 295

III. On Affections of the Testicle .. .. .. .. .. .. .. 300

IV. On Symptomatic Abscesses .. .. .. .. .. .. .. .. 303

V. On Prolapsus of the Rectum .. .. .. .. .. .. .. 305

VI. On Traumatic Delirium .. .. .. .. .. .. .. .. 306

VII. Fractures and Luxations of the Foot .. .. .. .. .. .. 308

VIII. Aneurism of the Brachial Artery .. .. .. .. .. .. 315

IX. Fracture of the Patella .. .. .. .. .. .. .. .. 316

X. Fractures of the Extremities .. .. .. .. .. .. .. 317

XI. Excision of Hæmorrhoidal Tumours .. .. .. .. .. .. 318

II.

Elements of Materia Medica and Therapeutics. By A. T. THOMSON, M.D. .. .. 321

III.

Elements of Surgery. By Robert LISTON, Esq. .. .. .. .. .. .. .. .. .. 328

IV.

Medico-Chirurgical Transactions, Vol. XVII.—Observations on Tumours. By Mr. LAWRENCE .. .. .. .. .. .. .. .. .. .. .. .. .. .. .. .. 333

V.

On some Effects of Inflammation of the Membranous Lining of the Larynx, &c. By Mr. WOOD .. .. .. .. .. .. .. .. .. .. .. .. .. .. 343

VI.

An Essay on the Structure and Functions of the Skin. By W. WOOD, M.D. .. 350

VII.

Experimental Investigation of the Effects of Loss of Blood. By MARSH. HALL, MD. 354

VIII.

The Uterus and the Human Ovum, during the first months of Pregnancy. By Dr. B. W. SEILER .. .. .. .. .. .. .. .. .. .. .. .. .. . .. 358

IX.

Cyclopædia of Practical Medicine. Dr. GILKREST on Yellow Fever .. .. .. 362

X.

MORBID ANATOMY.

1. Illustrations of the Elementary forms of Disease. By ROBERT CARSWELL, M.D. Fas. I.
2. Principles and Illustrations of Morbid Anatomy, &c. By J. HOPE, MD. Part II. } 366

XI.

Memoir of the Life and Writings of the Late Dr. Armstrong. By F. BOOTT, MD. 374

XII.

A Demonstration of the Nerves of the Human Body. Part III. Cerebral Nerves. By JOSEPH SWAN .. .. .. .. .. .. .. .. .. .. .. .. .. .. .. 385

XIII.

Memoirs of the Royal Academy of Medicine, Volume the Second .. .. .. .. 387
1. Ricord on some facts connected with Syphilis .. .. .. .. .. .. .. .. 388

XIV.

Nouveaux Elemens, &c. New Elements of Operative Medecine. By M. VELPEAU 395

XV.

Traité Pratique des Maladies de l'Uterus et de ses Annexes, &c. Practical Treatise on Diseases of the Uterus, &c. .. .. .. .. .. .. .. .. .. .. .. 406

XVI.

Sir GEO. BALLINGALL's Lectures on Military Surgery .. .. .. .. .. .. .. 428

### PERISCOPE.

1. Dr. Macfarlane's Clinical Reports from the Royal Infirmary of Glasgow—(Wounds of the Bladder—Contusions of the Urethra—Urinary Abscess—Abscess of Prostate Gland—Prolapsus Ani—Enlargements of Clitoris and Nymphæ—Imperforate Vagina, &c.) .. .. .. .. .. .. .. .. .. .. 443
2. Remarkable case of Empyema, with Operation. By Mr. Brigham of Manchester 450
3. Wound of the Heart—Patient surviving a month.. .. .. .. .. .. .. .. 453
4. New Theory of the Sounds of the Heart .. .. .. .. .. .. .. .. .. .. 454
5. Reclamation on the above subject .. .. .. .. .. .. .. .. .. .. .. 455
6. On Spontaneous Rupture of the Heart .. .. .. .. .. .. .. .. .. .. 455
7. Curious Malformations and Diseases of the Heart .. .. .. .. .. .. 456
8. Pericarditis, with Remarks .. .. .. .. .. .. .. .. .. .. .. .. 457
9. Identity of Magnetism and Electricity .. .. .. .. .. .. .. .. .. 458
10. Inversio Vesicæ .. .. .. .. .. .. .. .. .. .. .. .. .. .. .. 459
11. On Permanent Flexion of the Fingers .. .. .. .. .. .. .. .. .. 461
12. Diffused Inflammation after Bleeding .. .. .. .. .. .. .. .. .. 462
13. Cases of Hernia, without External Swelling .. .. .. .. .. .. .. 463
14. Dr. Thwaites on Pericarditis .. .. .. .. .. .. .. .. .. .. .. 464
15. Cutaneous Eruption in Cholera .. .. .. .. .. .. .. .. .. .. .. 466
16. Treatment of Cholera in France .. .. .. .. .. .. .. .. .. .. .. 466
17. Cancer of Stomach, without Symptoms.. .. .. .. .. .. .. .. .. .. 467
18. Inflammation of Spermatic Cord mistaken for Hernia .. .. .. .. .. 468
19. Remarkable Recovery of Motion in a Stiff-Joint .. .. .. .. .. .. .. 468
20. Organic Disease of Stomach, without Symptoms during Life—Sir P. Dun's Hospital .. .. .. .. .. .. .. .. .. .. .. .. .. .. .. .. .. 469

21. A Sketch of Broussais's Doctrine drawn by himself .. .. .. .. .. .. .. .. 469

22. Death of M. Portal .. .. .. .. .. .. .. .. .. .. .. .. .. .. .. .. 475

23. On the External Use of Water as a Remedy .. .. .. .. .. .. .. .. .. 476

24. Aneurism of the Gluteal Artery .. .. .. .. .. .. .. .. .. .. .. .. .. 476

25. Dr. Graves on Ulceration of the Stomach .. .. .. .. .. .. .. .. .. .. 477

26. Baron Alibert on the Cause of Epidemics .. .. .. .. .. .. .. .. .. .. 477

27. Antimony in Pneumonia in the Necker Hospital.. .. .. .. .. .. .. .. .. 478

28. Dr. Graves on Periostitis .. .. .. .. .. .. .. .. .. .. .. .. .. .. 481

29. Sir Charles Bell on Fracture of Patella .. .. .. .. .. .. .. .. .. .. 482

30. Clot Bey on the Guinea-Worm .. .. .. .. .. .. .. .. .. .. .. .. .. 483

31. Mr. Adams on Congenital Hernia .. .. .. .. .. .. .. .. .. .. .. .. 486

32. Spontaneous Cure of Hydrocele .. .. .. .. .. .. .. .. .. .. .. .. .. 489

33. On the Effects of smoking Tobacco .. .. .. .. .. .. .. .. .. .. .. 489

34. Composition of the Blood in Jaundice. By M. Lecanu .. .. .. .. .. .. 490

35. Dr. Davis's Obstetric Medicine.

I. Influence of the Moon on Menstruation .. .. .. .. .. .. .. .. 491

II. Nature of the Catamenial Discharge .. .. .. .. .. .. .. .. .. 492

III. Comparative Value of Life in the Male and Female Sex .. .. .. .. 493

36. Dr. M. Hall on Hibernation .. .. .. .. .. .. .. .. .. .. .. .. .. .. 497

37. On the March of Epidemics .. .. .. .. .. .. .. .. .. .. .. .. .. .. 500

38. Dr. Wolff on the Radix Caincæ .. .. .. .. .. .. .. .. .. .. .. .. .. 501

39. Dr. Wolff's Case of total Adhesion of Pericardium to the Heart.. .. .. .. 501

40. Dr. Macrobin on Insanity .. .. .. .. .. .. .. .. .. .. .. .. .. .. 502

41. Cases of Aneurism by Anastomosis .. .. .. .. .. .. .. .. .. .. .. .. 505

42. Dr. Macfarlane on Injuries of the Head .. .. .. .. .. .. .. .. .. .. 507

43. Mr. Middlemore on Ophthalmia Neonatorum .. .. .. .. .. .. .. .. .. 511

44. Dr. Chauffard on Issues and other Drains .. .. .. .. .. .. .. .. .. .. 512

45. Aphorism of Galen .. .. .. .. .. .. .. .. .. .. .. .. .. .. .. .. 514

46. Analogy between the Epidemic Cholera and the Sweating Sickness .. .. 514

47. Cysts in Thorax and Abdomen .. .. .. .. .. .. .. .. .. .. .. .. .. 514

48. Chemical Pathology of the Blood in Cholera .. .. .. .. .. .. .. .. .. 515

49. Case of Morbus Cæruleus.. .. .. .. .. .. .. .. .. .. .. .. .. .. .. 516

50. Dr. Prus on Pulmonary Emphysema .. .. .. .. .. .. .. .. .. .. .. .. 516

51. Dr. Forget on Air in the Blood .. .. .. .. .. .. .. .. .. .. .. .. .. 517

52. Assassination of M. Delpech .. .. .. .. .. .. .. .. .. .. .. .. .. .. 519

53. M. Humboldt on Goitre .. .. .. .. .. .. .. .. .. .. .. .. .. .. .. 520

54. On the Epidemic Sweating Fever at Aux-le Chateau .. .. .. .. .. .. .. 521

55. Memoir on the Functions of the Encephalon. By M. Rolando.. .. .. .. .. 522

56. External Use of Cyanuret of Potassium in Neuralgia .. .. .. .. .. .. . 525

57. Cold Affusions in Tetanus .. .. .. .. .. .. .. .. .. .. .. .. .. .. 526

58. Laceration of Perineum cured.. .. .. .. .. .. .. .. .. .. .. .. .. .. 526

59. Cyanuret of Mercury in Syphilis .. .. .. .. .. .. .. .. .. .. .. .. .. 527

60. Phlegmonous Erysipelas, treated by large Blisters .. .. .. .. .. .. .. 527

61. M. Autenrieth on Pertussis .. .. .. .. .. .. .. .. .. .. .. .. .. .. 527

62. Sugar as an Antidote to Poisoning by Copper .. .. .. .. .. .. .. .. .. 528

63. Clot Bey on Elephantiasis .. .. .. .. .. .. .. .. .. .. .. .. .. .. 529

64. Variolous Epidemic at Turin .. .. .. .. .. .. .. .. .. .. .. .. .. .. 529

65. Subcarbonate of Iron in Gastralgia .. .. .. .. .. .. .. .. .. .. .. .. 530

66. Cases from Dupuytren's "Clinique" .. 530
67. Poisoning by Sulphuric Acid .. 530
68. Fractures of the Radius mistaken .. 530
69. Remarkable instance of Female Precocity .. 530
70. Short-windedness in Horses .. 531
71. Insanity in France .. 531
72. Fracture of Bone by Action of Muscle .. 531
73. Death of Dr. Spurzheim .. 532
74. Clinique of M. Bouillaud .. 532
75. Foreign Body in the Trachea for Five Months .. 535
76. Alibert on Epidemics, (continued from p. 477) .. 535
77. Dr. Morgan's Lecture on Tetanus.. .. 536
78. Mr. Caswall on the Phenomena of Hearing .. 537
79. Singular Affection of the Organ of Language. By Dr. Gregory .. 538
80. INSTITUTE OF FRANCE.
1. Academy of Sciences .. 541
2. Academy of Medicine .. 543
81. Calculus extracted by Weiss's Forceps .. 547
82. Mr. Mayo on Loss of Memory from Age or Accident .. 548
83. Dr. Webster's Cases of Aphonia .. 551
84. Sir Charles Bell on the Organs of Voice.. .. 552
Of Articulation .. 555
85. History of the Glasgow Royal Infirmary. By Dr. M. S. Buchanan .. 559
86. Dr. Billing on the Sounds of the Heart .. 564
87. St. George's Hospital.
On the Causes of Death from Lithotomy .. 564
Abscess of the Pelvis .. 571
88. Surgical Account of the Siege of Antwerp .. 578
Contusions .. 579
Amaurosis from Contusions of the Eye-ball .. 579
89. Mr. M'Carthy's Miniature Bust of Mr. Brookes .. 581
90. Medical Reform—College of Physicians.. .. 582
BIBLIOGRAPHICAL RECORD.. .. 584

